# IL-18 on the rise: tracking inflammatory drift in hemodialysis patients - a pilot study

**DOI:** 10.1590/2175-8239-JBN-2025-0310en

**Published:** 2026-03-30

**Authors:** Jessyca Sousa de Brito, Hugo de Luca Corrêa, Thiago Santos Rosa, Aline Figueiredo, Carlos Eduardo H. V. P. F. Braga, Denise Mafra

**Affiliations:** 1 Universidade Federal do Rio de Janeiro, Programa de Pós-Graduação em Ciências Biológicas (Fisiologia), Rio de Janeiro, RJ, Brazil.; 2 Universidade Católica de Brasília, Programa de Pós-Graduação em Educação Física, Brasília, DF, Brazil.; 3 Universidade Católica de Brasília, Programa de Pós-Graduação em Ciência Genômica e Biotecnologia, Brasília, DF, Brazil.; 4 Universidade Estadual do Norte do Paraná, Programa de Pós-Graduação em Ciências do Movimento Humano, Jacarezinho, PR, Brazil.; 5 Universidade Federal Fluminense, Programa de Pós-Graduação em Ciências da Nutrição, Niterói, RJ, Brazil.; 6 Universidade Federal Fluminense, Programa de Pós-Graduação em Ciências Médicas, Niterói, RJ, Brazil.

**Keywords:** Renal Insufficiency, Chronic, Hemodialysis, Inflammation, Interleukin-18

## Abstract

**Introduction:**

Interleukin-18 (IL-18) is a pro-inflammatory cytokine associated with inflammaging and cellular senescence in chronic kidney disease (CKD).

**Objective:**

To evaluate longitudinal changes in plasma IL-18 levels in incident hemodialysis (HD) patients.

**Methods:**

An observational longitudinal study was conducted, with blood and anthropometric data collected at baseline, 6, and 12 months. IL-18 was measured by ELISA, and biochemical parameters were measured using Bioclin® kits.

**Results:**

Twenty-four patients (61.4 ± 3.5 years) were enrolled; 10 and 5 completed the 6- and 12-month assessments, respectively. IL-18 levels increased significantly at 6 months (p = 0.04) and at 12 months (p = 0.01) versus baseline and correlated positively with creatinine (r = 0.893; p = 0.04) and urea (r = 0.823; p = 0.05).

**Conclusion:**

In this pilot study with incident HD patients, plasma IL-18 increased over one year of treatment and was associated with uremia-related markers.

## Introduction

Chronic kidney disease (CKD) affects around 10% of the global population, representing a significant public health challenge^1^. The stages of CKD are classified according to the decline in kidney function and the degree of kidney damage. Initiation of dialysis is associated with increased inflammation, as evidenced by elevated interleukin-6 (IL-6) and C-reactive protein (CRP)^2,3^. Chronic low-grade inflammation in patients undergoing hemodialysis (HD) contributes to cardiovascular disease, protein-energy wasting, and frailty, a condition that overlaps with the concept of “inflammaging^4^.”

Evidence indicates that interleukin-18 (IL-18), also known as interferon-gamma-inducing factor, plays a relevant role in the pathophysiology of CKD, reinforcing its potential as a diagnostic and prognostic biomarker. IL-18 functions as a potent pro-inflammatory cytokine by inducing the synthesis of various inflammatory mediators, including immediate-response factors such as tumor necrosis factor-alpha (TNF-α) and interleukin-1 beta (IL-1β). Elevated plasma IL-18 levels have been identified as a hallmark of a subset of autoinflammatory conditions collectively referred to as “IL-18opathies,” in which IL-18 dysregulation plays a central pathogenic role^5^. Indeed, IL-18 is considered a predictor of death in patients with cardiovascular disease (CVD), as the induced production of IFN-*𝛾* leads to increased adhesion molecules and the formation of atherosclerotic plaque. In addition, emerging evidence suggests that persistently elevated IL-18 levels may contribute to the activation of senescence pathways by promoting oxidative stress, mitochondrial dysfunction, and chronic inflammatory responses, ultimately disrupting tissue homeostasis and impairing the function of immune and endothelial cells. This pro-senescent environment may accelerate the development of age-related comorbidities such as atherosclerosis, sarcopenia, and frailty, which are commonly observed in hemodialysis patients^6^. The present study aimed to evaluate IL-18 levels at the beginning of dialysis treatment and after 6 and 12 months and to investigate possible associations with other inflammatory biomarkers and clinical parameters.

## Methods

### Study Design and Patients

This study was conducted at the Prodoctor Hemodialysis Clinic, RJ, Brazil, between August 2023 and August 2024. Incident hemodialysis patients (with up to 30 days of dialysis) were invited to participate. A non-probabilistic convenience sample was used for this study.

The study was approved by the Ethics Committee of the School of Medicine of *Universidade Federal Fluminense* (protocol number 6.246.394). All patients were informed about the study and signed an informed consent form before the intervention. Patients of both sexes, aged 18 years or older, who had recently initiated hemodialysis (defined as being on HD for up to 30 days) with either a fistula or catheter as vascular access were considered eligible for inclusion. Patients with a history of kidney transplantation, active infectious diseases, previous hemodialysis treatment, current smoking, or illicit drug use were excluded.

This pilot study included patients receiving maintenance hemodialysis with high-flux synthetic membrane dialyzers. Dialysis was conducted under standardized conditions, with blood flow rates typically ranging from 300 to 400 mL/min and a dialysate flow of approximately 500 mL/min. Treatments were performed three times weekly, each lasting approximately 4 hours. Dialysis adequacy was assessed only at baseline, with a median Kt/V of 1.4.

### Blood Collection and Biochemical Analyses

Blood samples were collected before the start of the hemodialysis session to avoid acute dialysis-related effects on circulating biomarkers. Samples were collected in Vacutainer tubes, centrifuged at 3500 rpm for 15 minutes at 4°C to separate plasma and serum, and then stored at –80°C. Biochemical parameters were determined using commercial Bioclin® kits on an automatic biochemical analyzer. Plasma IL-18 levels were measured in triplicate using ELISA kits (R&D Systems, Minneapolis, MN), following the manufacturer’s instructions.

### Anthropometric Measurements

Anthropometric measurements were performed by a trained researcher using standardized procedures. Body weight (dry weight) and height were measured with calibrated equipment, and body mass index (BMI) was calculated as weight (Kg) divided by height squared (m^2^). Skinfold thicknesses (bicipital, tricipital, subscapular, and suprailiac) were measured with a Lange Skinfold Caliper (1-mm precision; Cambridge Scientific Industries Inc.). Waist circumference was measured with a non-elastic tape at the midpoint between the lowest rib and the iliac crest at the end of a normal expiration. Body density was estimated from the sum of the four skinfold measurements using the equations proposed by Durnin and Womersley^7^. Body fat percentage was calculated from body density using the Siri equation^8^. Fat-free mass was obtained by subtracting fat mass from total body weight.

### Statistical Analysis

Descriptive data were expressed as means and standard deviations. Normality of the data distribution was assessed using the Shapiro–Wilk test. Comparisons of clinical, biochemical, and anthropometric parameters across the three time points (baseline, 6 months, and 12 months) were performed using repeated-measures one-way analysis of variance (ANOVA). When significant differences were detected, *post hoc* analyses were conducted using the Bonferroni correction to identify specific time-point differences. Pearson’s correlation coefficient was calculated to explore the relationship between IL-18 levels and other clinical variables. Statistical significance was set at *p* < 0.05. All analyses were performed using GraphPad Prism 10 (GraphPad Software, San Diego, CA, USA).

## Results

Initially, 24 patients were recruited, and their data were evaluated at baseline. Of these, 10 patients completed the 6-month follow-up, and five completed the 12-month assessment. The remaining participants had not yet reached the 6- or 12-month time points and were therefore excluded from the final longitudinal analysis. During follow-up, nine patients discontinued participation: five died (three after baseline and two after 12 months), two were transferred to another dialysis unit (after baseline), one underwent a kidney transplant (after 12 months), and one withdrew due to cancer treatment and clinical deterioration.

**Figure 1:**
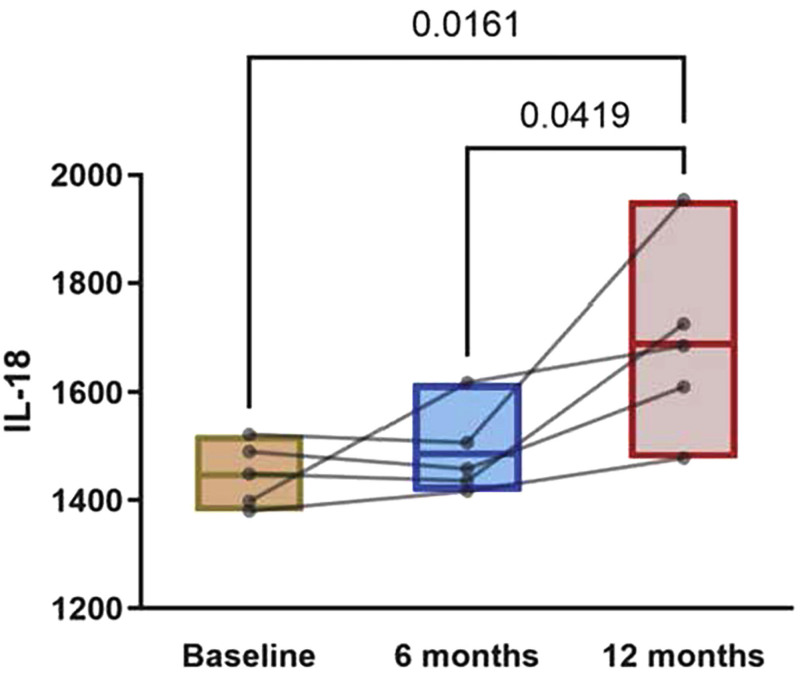
Plasma IL-18 levels in CKD patients at baseline, 6 months, and 12 months of HD treatment.

The average age was 61.4 ± 3.5 years. Systemic arterial hypertension was observed in 60% of patients, and all patients were diagnosed with diabetes mellitus. Table 1 presents the anthropometric and biochemical parameters of these patients. Figure 1 illustrates the increased levels of IL-18 following the initiation of HD treatment.

**Table 1:** Anthropometric and biochemical data of patients with ckd at baseline, 6 months, and 12 months after the start of hemodialysis treatment

Parameters	Baseline (n = 5)	6 months of HD (n = 5)	12 months of HD (n = 5)	p-values
Anthropometric Parameters				
Body mass index (kg/m^2^)	23.8 ± 8.5	24.1 ± 8.0	24.5 ± 8.0	0.55
Waist circumference (cm)	89.2 ± 23.7	77.7 ± 6.7	90.0 ± 23.0	0.50
Body fat (%)	23.8 ± 11.1	18.0 ± 2.5	24.3 ± 9.8	0.54
Fat free mass (Kg)	53.9 ± 12.0	50.9 ± 5.2	55.6 ± 12.2	0.80
Biochemical Parameters				
Urea (mg/dL)	143.6 ± 33.1	120 ± 66.6	132.4 ± 58.8	0.79
Creatinine (mg/dL)	7.0 ± 2.0	6.9 ± 3.9	8.0 ± 3.4	0.77
Albumin (g/dL)	3.7 ± 0.3	3.9 ± 0.2	3.8 ± 0.4	0.68
Glucose (mg/dL)	177.4 ± 100.9	200.0 ± 130.8	210.4 ± 117.2	0.90
Calcium (mg/dL)	8.4 ± 0.5	8.5 ± 0.8	7.8 ± 0.6	0.22
Phosphorus (mg/dL)	5.4 ± 1.5	4.4 ± 1.8	4.3 ± 1.6	0.51
Magnesium (mg/dL)	1.9 ± 0.3	1.9 ± 0.2	1.8 ± 0.2	0.77
Total Cholesterol (mg/dL)	154.6 ± 32.7	158.2 ± 35.3	157.4 ± 36.6	0.98
High-Density Lipoprotein (mg/dL)	43.4 ± 18.8	37.2 ± 12.6	36.6 ± 14.5	0.75
Low-Density Lipoprotein (mg/dL)	69.1 ± 15.7	75.8 ± 27.3	77.2 ± 35.4	0.88
Triglyceride (mg/dL)	210.6 ± 176.0	226.2 ± 140.1	218.0 ± 160.2	0.98
C-reactive protein (mg/L)	29.6 ± 25.1	8.2 ± 7.6	18.6 ± 17.5	0.21
Interleukin-18 (pg/mL)	1447.8 ± 59.9	1487.2 ± 80.5	1691.0 ± 174.9	**0.01**

Additionally, IL-18 exhibited a strong positive correlation with creatinine (r = 0.893; p = 0.041) and urea (r = 0.823; p = 0.050).

## Discussion

This study investigated longitudinal changes in IL-18 levels during the first year of hemodialysis and found a significant increase after 12 months, accompanied by correlations with markers of uremia.

IL-18 belongs to the IL-1 superfamily and functions as a potent proinflammatory cytokine, playing a central role in the induction of Th1-type immune responses. Intracellularly, IL-18 is initially synthesized as a biologically inactive precursor of approximately 24 kDa. Its activation occurs through enzymatic cleavage by caspase-1, yielding the mature and bioactive 18 kDa form, which is subsequently secreted into the extracellular environment^9^. Several intracellular signaling molecules are activated upon IL-18 binding to the interleukin-18 receptor (IL-18R), such as myeloid differentiation primary response protein 88 (MyD88), the nuclear factor *kappa-B* (NF-κB) transcription pathway, interleukin-1 receptor-associated kinase, and the mitogen-activated protein kinase (MAPK) pathway^10^. Therefore, IL-18 can be regarded as a key cytokine exacerbating inflammatory conditions. Indeed, a review has described the possible clinical roles of IL-18, as it induces IFN-γ synthesis, and high levels have been referred to as “IL-18opathies^5^.”

Elevated IL-18 concentrations have been consistently reported in patients with CKD and in those undergoing hemodialysis, where they are associated with cardiovascular risk and adverse clinical outcomes. Previous studies have demonstrated that IL-18 predicts hospitalization and mortality in HD patients and is strongly linked to arterial stiffness and vascular dysfunction, suggesting a potential causal role in endothelial injury rather than a mere marker of systemic inflammation^11,12^.

A study conducted by Formanowicz *et al*. (2015) investigated IL-18 as a predictor of CVD mortality in CKD patients with a history of acute myocardial infarction (AMI). The results indicated that elevated IL-18 levels (above 1584.5 pg/mL) were significantly associated with an increased risk of CVD mortality, with a 20.63-fold increase compared to patients with lower levels. Moreover, IL-18 was found to be a superior prognostic marker compared with other traditional indicators such as high-sensitivity CRP, carotid intima-media thickness (CIMT), and N-terminal prohormone of brain natriuretic peptide (NT-proBNP)^13^.

Arapi *et al*. (2018) demonstrated increased IL-18 mRNA expression in patients with carotid artery stenosis, suggesting its involvement in atherosclerosis-related inflammation^14^. IL-18-binding protein (IL-18BP) expression was higher in symptomatic patients, indicating a possible link with clinical manifestations. Additionally, a trend toward an association between IL-18 levels and stenosis severity was observed, while the IL-18-137 G/C polymorphism showed no significant association^14^.

Eltawab Radwan *et al*. (2024) showed that serum IL-18 concentrations were significantly higher in patients with CKD undergoing HD than in those with acute kidney injury (AKI), irrespective of sex. Furthermore, IL-18 levels were positively correlated with age in both groups. These findings suggest that IL-18 may serve as a valuable biomarker for distinguishing CKD from AKI and for reflecting age-related changes^15^.

Continuous monitoring of inflammatory markers may help predict clinical outcomes and identify at-risk patients early. The international MONitoring Dialysis Outcomes (MONDO) Consortium study, which includes hemodialysis databases from 31 countries in Europe, North America, South America, and Asia, analyzed the dynamics of inflammatory markers (total white blood cell count, neutrophil count, lymphocyte count, serum albumin, and CRP) in patients who initiated hemodialysis over 36 months. Interestingly, the results indicated that the levels of these markers decrease after the initiation of hemodialysis, remain stable in survivors, but increase again approximately 6 months before death^16^.

Beyond its role as a marker of systemic inflammation and uremic burden, IL-18 has been increasingly associated with processes of premature cellular senescence and inflammaging, especially in chronic disease states such as CKD^17^. Persistent elevation of IL-18 has been linked to oxidative stress, mitochondrial dysfunction, and immunosenescence, all of which contribute to the accelerated aging phenotype frequently observed in hemodialysis patients.

This short communication reports a progressive increase in plasma IL-18 levels over one year of hemodialysis treatment and presents exploratory associations with creatinine and urea. Given the small sample size and loss to follow-up, these findings should be interpreted with caution and considered hypothesis-generating, as they may introduce selection bias and limit generalizability. In addition, the lack of systematic information on dietary intake and Kt/V may influence inflammatory pathways, further limiting the interpretation of this study and potentially introducing residual confounding. Nonetheless, the results underscore the potential relevance of IL-18 as a marker of inflammatory burden in this population, supporting its monitoring in hemodialysis patients and highlighting the need for larger longitudinal studies to elucidate its prognostic value.

## Data Availability

Data will be made available upon request.

## References

[1] Romagnani P, Agarwal R, Chan JCN, Levin A, Kalyesubula R, Karam S (2025). Chronic kidney disease. Nat Rev Dis Primers.

[2] Kadatane SP, Satariano M, Massey M, Mongan K, Raina R (2023). The role of inflammation in CKD. Cells.

[3] Sahib A, Choudhury C, Wani IA, Wani MM (2024). Evaluation of inflammatory status in chronic kidney disease
patients and a comparison between hemodialysis and peritoneal dialysis
patients. Cureus.

[4] Cobo G, Lindholm B, Stenvinkel P (2018). Chronic inflammation in end-stage renal disease and
dialysis. Nephrol Dial Transplant.

[5] Landy E, Carol H, Ring A, Canna S (2024). Biological and clinical roles of IL-18 in inflammatory
diseases. Nat Rev Rheumatol.

[6] Martini H, Passos JF (2023). Cellular senescence: all roads lead to
mitochondria. FEBS J.

[7] Durnin JV, Womersley J (1974). Body fat assessed from total body density and its estimation from
skinfold thickness: measurements on 481 men and women aged from 16 to 72
years. Br J Nutr.

[8] Siri WE, Brozek J, Hanschel A (1961). Techniques for measuring body composition.

[9] Hirooka Y, Nozaki Y (2021). Interleukin-18 in inflammatory kidney disease. Front Med.

[10] Yasuda K, Nakanishi K, Tsutsui H (2019). Interleukin-18 in health and disease. Int J Mol Sci.

[11] Chiang CK, Hsu SP, Pai MF, Peng YS, Ho TI, Liu SH (2004). Interleukin-18 is a strong predictor of hospitalization in
haemodialysis patients. Nephrol Dial Transplant.

[12] Porazko T, Kuz´niar J, Kusztal M, Kuz´niar TJ, Weyde W, Kuriata-Kordek M (2008). IL-18 is involved in vascular injury in end-stage renal disease
patients. Nephrol Dial Transplant.

[13] Formanowicz D, Wanic-Kossowska M, Pawliczak E, Radom M, Formanowicz P (2015). Usefulness of serum interleukin-18 in predicting cardiovascular
mortality in patients with chronic kidney disease-systems and clinical
approach. Sci Rep.

[14] Arapi B, Bayogˇlu B, Cengiz M, Dirican A, Deser SB, Junusbekov Y (2018). Increased expression of interleukin-18 mrna is associated with
carotid artery stenosis. Balkan Med J.

[15] Radwan GAE, El-Said Yousef A, Bayomy MF (2024). Serum interleukin 18 level in kidney diseases and
age. Urol Ann.

[16] Yousif DE, Ye X, Stuard S, Berbessi J, Guinsburg AM, Usvyat LA (2023). Biphasic dynamics of inflammatory markers following hemodialysis
initiation: results from the international monitoring dialysis outcome
initiative. Kidney Int Rep.

[17] Kooman JP, Kotanko P, Schols AMWJ, Shiels PG, Stenvinkel P (2014). Chronic kidney disease and premature ageing. Nat Rev Nephrol.

