# Supplementary material for: IL-18 on the rise: tracking inflammatory drift in hemodialysis patients - a pilot study
Source: J Bras Nefrol. 2026 Mar 30;48(2):e20250310. doi: 10.1590/2175-8239-JBN-2025-0310en (PMC13034619; doi:10.1590/2175-8239-JBN-2025-0310en)
Supplement: Translation

# Translation: IL-18 em elevação: acompanhando a progressão do estado inflamatório em pacientes em hemodiálise - estudo piloto

**DOI:** 10.1590/2175-8239-JBN-2025-0310pt

**Authors:** Jessyca Sousa de Brito, Hugo de Luca Corrêa, Thiago Santos Rosa, Aline Figueiredo, Carlos Eduardo H. V. P. F. Braga, Denise Mafra

**Affiliations:** 1 Universidade Federal do Rio de Janeiro, Programa de Pós-Graduação em Ciências Biológicas (Fisiologia), Rio de Janeiro, RJ, Brasil.; 2 Universidade Católica de Brasília, Programa de Pós-Graduação em Educação Física, Brasília, DF, Brasil.; 3 Universidade Católica de Brasília, Programa de Pós-Graduação em Ciência Genômica e Biotecnologia, Brasília, DF, Brasil.; 4 Universidade Estadual do Norte do Paraná, Programa de Pós-Graduação em Ciências do Movimento Humano, Jacarezinho, PR, Brasil.; 5 Universidade Federal Fluminense, Programa de Pós-Graduação em Ciências da Nutrição, Niterói, RJ, Brasil.; 6 Universidade Federal Fluminense, Programa de Pós-Graduação em Ciências Médicas, Niterói, RJ, Brasil.

**Keywords:** Insuficiência Renal Crônica, Hemodiálise, Inflamação, Interleucina-18

## Abstract

**Introdução:**

A interleucina-18 (IL-18) é uma citocina pró-inflamatória associada ao *inflammaging* e à senescência celular na doença renal crônica (DRC).

**Objetivo:**

Avaliar as alterações longitudinais nos níveis plasmáticos de IL-18 em pacientes incidentes em hemodiálise (HD).

**Métodos:**

Foi conduzido um estudo clínico observacional longitudinal, com coleta de sangue e de dados antropométricos no momento basal, aos 6 e aos 12 meses. A IL-18 foi quantificada por ELISA, e os parâmetros bioquímicos foram analisados com kits Bioclin®.

**Resultados:**

Vinte e quatro pacientes (61,4 ± 3,5 anos) foram incluídos no estudo; 10 completaram as avaliações aos 6 meses de HD e 5 pacientes, após 1 ano. Os níveis de IL-18 aumentaram significativamente aos 6 meses (p = 0,04) e aos 12 meses (p = 0,01) e apresentaram correlação positiva com creatinina (r = 0,893; p = 0,04) e ureia (r = 0,823; p = 0,05).

**Conclusão:**

Neste estudo piloto com pacientes incidentes em HD, os níveis plasmáticos de IL-18 aumentaram ao longo de um ano de tratamento e estiveram associados a marcadores relacionados à uremia.

## Introdução

A doença renal crônica (DRC) afeta cerca de 10% da população mundial e representa um importante desafio de saúde pública^1^. Os estágios da DRC são classificados de acordo com o declínio da função renal e o grau de dano renal. O início da diálise está associado ao aumento da inflamação, evidenciado por níveis elevados de interleucina-6 (IL-6) e de proteína C-reativa (PCR)^2,3^. A inflamação crônica de baixo grau em pacientes em hemodiálise (HD) contribui para o desenvolvimento de doenças cardiovasculares, desnutrição energético-proteica e fragilidade, condição que se sobrepõe ao conceito de *inflammaging* ^4^.

Evidências indicam que a interleucina-18 (IL-18), também conhecida como fator indutor de interferon-gama, desempenha papel relevante na fisiopatologia da DRC, reforçando seu potencial como biomarcador diagnóstico e prognóstico. A IL-18 atua como potente citocina pró-inflamatória ao induzir a síntese de diversos mediadores inflamatórios, incluindo fatores de resposta imediata, como o fator de necrose tumoral alfa (TNF-α) e a interleucina-1 beta (IL-1β). Níveis plasmáticos elevados de IL-18 têm sido identificados como característica marcante de um subconjunto de condições autoinflamatórias, coletivamente denominadas *“IL-18opatias”*, nas quais a desregulação da IL-18 desempenha papel patogênico central^5^. De fato, a IL-18 é considerada um preditor de mortalidade em pacientes com doenças cardiovasculares, uma vez que a produção induzida de IFN-γ leva ao aumento da expressão de moléculas de adesão e à formação de placas ateroscleróticas. Além disso, evidências emergentes sugerem que níveis persistentemente elevados de IL-18 podem contribuir para a ativação de vias de senescência ao promover estresse oxidativo, disfunção mitocondrial e respostas inflamatórias crônicas, culminando na ruptura da homeostase tecidual e no comprometimento da função de células imunes e endoteliais. Esse ambiente pró-senescente pode acelerar o desenvolvimento de comorbidades relacionadas ao envelhecimento, como aterosclerose, sarcopenia e fragilidade, comumente observadas em pacientes em hemodiálise^6^. O presente estudo teve como objetivo avaliar os níveis de IL-18 no início do tratamento dialítico e após 6 e 12 meses, bem como investigar possíveis associações com outros biomarcadores inflamatórios e com parâmetros clínicos.

## Métodos

### Desenho do Estudo e Pacientes

Este estudo foi conduzido na Clínica de Hemodiálise Prodoctor (RJ, Brasil), entre agosto de 2023 e agosto de 2024. Pacientes incidentes em hemodiálise (com até 30 dias de tratamento dialítico) foram convidados a participar do estudo. Foi utilizada uma amostra de conveniência não probabilística.

O estudo foi aprovado pelo Comitê de Ética em Pesquisa da Faculdade de Medicina da Universidade Federal Fluminense (nº 6.246.394). Todos os pacientes foram devidamente informados sobre os objetivos do estudo e assinaram o Termo de Consentimento Livre e Esclarecido antes da intervenção. Foram considerados elegíveis para inclusão pacientes de ambos os sexos, com idade igual ou superior a 18 anos, que haviam iniciado recentemente a hemodiálise (definida como até 30 dias de tratamento), utilizando fístula ou cateter como acesso vascular. Pacientes com histórico de transplante renal, doenças infecciosas ativas, tratamento prévio por hemodiálise, tabagismo atual ou uso de drogas ilícitas foram excluídos.

Este estudo piloto incluiu pacientes em hemodiálise de manutenção utilizando dialisadores de membrana sintética de alto fluxo (*high-flux*). As sessões de diálise foram realizadas sob condições padronizadas, com fluxo sanguíneo geralmente entre 300 e 400 mL/min e fluxo de dialisato de aproximadamente 500 mL/min. O tratamento foi realizado três vezes por semana, com sessões de cerca de 4 horas. A adequação dialítica foi avaliada apenas no momento basal, com Kt/V médio de 1,4.

### Coleta de Sangue e Análises Bioquímicas

Amostras de sangue foram coletadas antes do início da sessão de hemodiálise para evitar efeitos agudos da diálise sobre os biomarcadores circulantes. As amostras foram coletadas em tubos *Vacutainer*, centrifugadas a 3500 rpm por 15 minutos a 4 °C para separar o plasma e o soro e, em seguida, armazenadas a –80 °C. Os parâmetros bioquímicos foram determinados com kits comerciais Bioclin® em um analisador bioquímico automático. Os níveis plasmáticos de IL-18 foram medidos em triplicata, utilizando kits ELISA (*R&D Systems*, Minneapolis, MN), conforme as instruções do fabricante.

### Medidas Antropométricas

As medidas antropométricas foram realizadas por um pesquisador treinado, seguindo procedimentos padronizados. O peso corporal (peso seco) e a altura foram medidos com equipamentos calibrados, e o índice de massa corporal (IMC) foi calculado como o peso (Kg) dividido pelo quadrado da altura (m^^2^^). As espessuras das dobras cutâneas (bicipital, tricipital, subescapular e suprailíaca) foram medidas com um adipômetro de *Lange* (precisão de 1 mm; *Cambridge Scientific Industries Inc.*). Já a circunferência da cintura foi medida com fita métrica não elástica, no ponto médio entre a última costela e a crista ilíaca, ao final de uma expiração normal. Estimou-se a densidade corporal a partir da soma das quatro medidas de pregas cutâneas, utilizando as equações propostas por Durnin e Womersley^7^. A porcentagem de gordura corporal foi calculada a partir da densidade corporal, utilizando a equação de Siri^8^. A massa livre de gordura foi obtida subtraindo-se a gordura do peso corporal total.

### Análise Estatística

Os dados descritivos foram expressos como médias e desvios-padrão. A normalidade da distribuição dos dados foi avaliada pelo teste de *Shapiro–Wilk*. As comparações dos parâmetros clínicos, bioquímicos e antropométricos nos três momentos avaliados (basal, 6 meses e 12 meses) foram realizadas por meio de análise de variância de uma via para medidas repetidas (*one-way repeated-measures ANOVA*). Quando identificadas diferenças significativas, análises *post hoc* foram conduzidas, com correção de *Bonferroni*, para identificar diferenças específicas entre os momentos. O coeficiente de correlação de *Pearson* foi utilizado para explorar a relação entre os níveis de IL-18 e outras variáveis clínicas. O nível de significância estatística foi estabelecido em p < 0,05. Todas as análises foram realizadas no software *GraphPad Prism* 10 (*GraphPad Software*, San Diego, CA, EUA).

## Resultados

Inicialmente, 24 pacientes foram recrutados, e seus dados foram avaliados no momento basal. Destes, 10 pacientes completaram o acompanhamento por 6 meses e 5 completaram a avaliação por 12 meses. Os demais participantes ainda não haviam atingido os pontos temporais de 6 ou 12 meses e, portanto, foram excluídos da análise longitudinal final. Durante o seguimento, nove pacientes descontinuaram a participação no estudo: cinco foram a óbito (três após a avaliação basal e dois após um ano), dois foram transferidos para outra unidade de diálise (após a avaliação basal), um foi submetido a transplante renal (após um ano) e um foi excluído devido ao início de tratamento oncológico e piora clínica.

A idade média dos pacientes foi de 61,4 ± 3,5 anos, com hipertensão arterial sistêmica observada em 60% dos pacientes; todos apresentavam diagnóstico de diabetes mellitus. Os parâmetros antropométricos e bioquímicos desses pacientes estão descritos na Tabela 1. A Figura 1 ilustra o aumento dos níveis de IL-18 após o início do tratamento com hemodiálise. Além disso, a IL-18 apresentou correlação positiva com os níveis de creatinina (r = 0,893; p = 0,041) e de ureia (r = 0,823; p = 0,050).

**Tabela 1:** Dados antropométricos e bioquímicos de pacientes com drc no momento basal, após 6 meses e 12 meses do início do tratamento hemodialítico

Parâmetros	Início (n = 5)	6 meses de HD (n = 5)	12 meses de HD (n = 5)	valor de p
Parâmetros Antropométricos				
Índice de massa corporal (kg/m^2^)	23,8 ± 8,5	24,1 ± 8,0	24,5 ± 8,0	0,55
Circunferência da cintura (cm)	89,2 ± 23,7	77,7 ± 6,7	90,0 ± 23,0	0,50
Gordura corporal (%)	23,8 ± 11,1	18,0 ± 2,5	24,3 ± 9,8	0,54
Massa livre de gordura (Kg)	53,9 ± 12,0	50,9 ± 5,2	55,6 ± 12,2	0,80
Parâmetros Bioquímicos				
Ureia (mg/dL)	143,6 ± 33,1	120 ± 66,6	132,4 ± 58,8	0,79
Creatinina (mg/dL)	7,0 ± 2,0	6,9 ± 3,9	8,0 ± 3,4	0,77
Albumina (g/dL)	3,7 ± 0,3	3,9 ± 0,2	3,8 ± 0,4	0,68
Glicose (mg/dL)	177,4 ± 100,9	200,0 ± 130,8	210,4 ± 117,2	0,90
Cálcio (mg/dL)	8,4 ± 0,5	8,5 ± 0,8	7,8 ± 0,6	0,22
Fósforo (mg/dL)	5,4 ± 1,5	4,4 ± 1,8	4,3 ± 1,6	0,51
Magnésio (mg/dL)	1,9 ± 0,3	1,9 ± 0,2	1,8 ± 0,2	0,77
Colesterol total (mg/dL)	154,6 ± 32,7	158,2 ± 35,3	157,4 ± 36,6	0,98
Lipoproteína de alta densidade (mg/dL)	43,4 ± 18,8	37,2 ± 12,6	36,6 ± 14,5	0,75
Lipoproteína de baixa densidade (mg/dL)	69,1 ± 15,7	75,8 ± 27,3	77,2 ± 35,4	0,88
Triglicerídeos (mg/dL)	210,6 ± 176,0	226,2 ± 140,1	218,0 ± 160,2	0,98
Proteína C-reativa (mg/L)	29,6 ± 25,1	8,2 ± 7,6	18,6 ± 17,5	0,21
Interleucina-18 (pg/mL)	1447,8 ± 59,9	1487,2 ± 80,5	1691,0 ± 174,9	**0,01**

**Figura 1:** Níveis plasmáticos de IL-18 em pacientes com DRC no momento basal, após 6 meses e 12 meses de tratamento hemodialítico.

## Discussão

Este estudo investigou as alterações longitudinais nos níveis de IL-18 durante o primeiro ano de hemodiálise e identificou um aumento significativo após 12 meses, acompanhado de correlações com marcadores de uremia.

A IL-18 pertence à superfamília da IL-1 e atua como potente citocina pró-inflamatória, desempenhando papel central na indução de respostas imunes do tipo Th1. Intracelularmente, a IL-18 é sintetizada como um precursor biologicamente inativo de aproximadamente 24 kDa. Sua ativação ocorre por meio da clivagem enzimática pela caspase-1, originando a forma madura e biologicamente ativa de 18 kDa, a qual é posteriormente secretada para o meio extracelular^9^. Diversas moléculas de sinalização intracelular são ativadas após a ligação da IL-18 ao receptor de interleucina-18 (IL-18R), incluindo a proteína de resposta primária à diferenciação mieloide 88 (MyD88), a via de transcrição do fator nuclear *kappa* B (NF-κB), a quinase associada ao receptor de interleucina-1 e as quinases ativadas por mitógenos (MAPK)^10^. Dessa forma, a IL-18 pode ser considerada uma citocina-chave na exacerbação de condições inflamatórias. De fato, uma revisão demonstrou os possíveis papéis clínicos da IL-18, uma vez que esta induz a síntese de IFN-γ, e níveis elevados podem ser caracterizados como *“IL-18opatias”* ^5^.

Concentrações elevadas de IL-18 têm sido consistentemente relatadas em pacientes com DRC em hemodiálise, associadas ao risco cardiovascular e a desfechos clínicos adversos. Estudos prévios demonstraram que a IL-18 prediz hospitalização e mortalidade em pacientes em HD e está fortemente relacionada à rigidez arterial e à disfunção vascular, sugerindo um possível papel causal na lesão endotelial, e não apenas como marcador de inflamação sistêmica^11,12^.

Um estudo conduzido por Formanowicz *et al*. (2015) investigou a IL-18 como preditor de mortalidade cardiovascular em pacientes com DRC e histórico de infarto agudo do miocárdio (IAM). Os resultados demonstraram que níveis elevados de IL-18 (acima de 1584,5 pg/mL) estiveram significativamente associados a um risco aumentado de mortalidade cardiovascular, representando um aumento de 20,63 vezes em comparação aos pacientes com níveis mais baixos. Além disso, a IL-18 mostrou-se um marcador prognóstico superior a outros indicadores tradicionais, como a PCR de alta sensibilidade, a espessura médio-intimal da carótida (CIMT) e o pró-hormônio N-terminal do peptídeo natriurético cerebral (NT-proBNP)^13^.

Arapi *et al.* (2018) demonstraram aumento da expressão de mRNA de IL-18 em pacientes com estenose da artéria carótida, sugerindo seu envolvimento na inflamação associada à aterosclerose^14^. A expressão da proteína ligante de IL-18 (IL-18BP) foi maior em pacientes sintomáticos, o que sugere possível associação com as manifestações clínicas. Adicionalmente, observou-se uma tendência de associação entre os níveis de IL-18 e a gravidade da estenose, enquanto o polimorfismo IL-18-137 G/C não apresentou associação significativa^14^.

Eltawab Radwan *et al*. (2024) demonstraram que as concentrações séricas de IL-18 foram significativamente mais elevadas em pacientes com DRC em hemodiálise, quando comparadas às observadas em pacientes com lesão renal aguda (LRA), independentemente do sexo. Além disso, os níveis de IL-18 apresentaram correlação positiva com a idade em ambos os grupos. Esses achados sugerem que a IL-18 pode atuar como um biomarcador útil para diferenciar DRC de LRA, bem como refletir alterações associadas ao envelhecimento^15^.

O monitoramento contínuo de marcadores inflamatórios pode auxiliar na predição de desfechos clínicos e na identificação precoce de pacientes em risco. O estudo do Consórcio Internacional *MONitoring Dialysis Outcomes* (MONDO), que reúne bancos de dados de hemodiálise de 31 países da Europa, da América do Norte, da América do Sul e da Ásia, analisou a dinâmica de marcadores inflamatórios (contagem total de leucócitos, contagem de neutrófilos, contagem de linfócitos, albumina sérica e PCR) em pacientes que iniciaram hemodiálise ao longo de 36 meses. Interessantemente, os resultados demonstraram que os níveis desses marcadores diminuem após o início da hemodiálise, permanecem estáveis entre os sobreviventes e voltam a aumentar aproximadamente seis meses antes do óbito^16^.

Além de seu papel como marcador de inflamação sistêmica e de carga urêmica, a IL-18 tem sido cada vez mais associada a processos de senescência celular precoce e de *inflammaging*, especialmente em estados de doença crônica, como a DRC^17^. A elevação persistente da IL-18 tem sido relacionada ao estresse oxidativo, à disfunção mitocondrial e à imunossenescência, fatores que contribuem para o fenótipo de envelhecimento acelerado frequentemente observado em pacientes em hemodiálise.

Esta comunicação breve relata um aumento progressivo dos níveis plasmáticos de IL-18 ao longo de um ano de tratamento hemodialítico e apresenta associações exploratórias com creatinina e ureia. Diante do pequeno tamanho amostral e das perdas durante o seguimento, esses achados devem ser interpretados com cautela e considerados geradores de hipóteses, uma vez que podem introduzir viés de seleção e limitar a generalização dos resultados. Além disso, a ausência de informações sistemáticas sobre a ingestão alimentar e o Kt/V pode influenciar as vias inflamatórias, o que delimita ainda mais o escopo deste estudo e, potencialmente, introduz confundimento residual. Ainda assim, os resultados ressaltam a potencial relevância da IL-18 como marcador da carga inflamatória nessa população, apoiando seu monitoramento em pacientes em hemodiálise e destacando a necessidade de estudos longitudinais maiores para elucidar seu valor prognóstico.

## Disponibilidade de Dados

Os dados estarão disponíveis mediante solicitação.
